# An approach toward public hospital performance assessment: Retraction

**DOI:** 10.1097/MD.0000000000009841

**Published:** 2018-02-02

The article, “An approach toward public hospital performance assessment”,^[1]^ which appeared in Volume 95, Issue 36 of *Medicine*, is being retracted due to a reporting error in the data. The reporting error invalidated the conclusion of the paper.
